# Implementation Challenges of 3D Printing in Prosthodontics: A Ranking-Type Delphi

**DOI:** 10.3390/ma15020431

**Published:** 2022-01-07

**Authors:** Klara Loges, Victor Tiberius

**Affiliations:** Faculty of Economics and Social Sciences, University of Potsdam, 14469 Potsdam, Germany; klara.loges@uni-potsdam.de

**Keywords:** 3D printing, prosthodontics, ranking type Delphi study, additive manufacturing, dentistry

## Abstract

The reduction in cost and increasing benefits of 3D printing technologies suggest the potential for printing dental prosthetics. However, although 3D printing technologies seem to be promising, their implementation in practice is complicated. To identify and rank the greatest implementation challenges of 3D printing in dental practices, the present study surveys dentists, dental technicians, and 3D printing companies using a ranking-type Delphi study. Our findings imply that a lack of knowledge is the most crucial obstacle to the implementation of 3D printing technologies. The high training effort of staff and the favoring of conventional methods, such as milling, are ranked as the second and third most relevant factors. Investment costs ranked in seventh place, whereas the lack of manufacturing facilities and the obstacle of print duration ranked below average. An inclusive implementation of additive manufacturing could be achieved primarily through the education of dentists and other staff in dental practices. In this manner, production may be managed internally, and the implementation speed may be increased.

## 1. Introduction

Three-dimensional (3D) printing, also known as additive manufacturing, was developed in the late 1980s and is the process of layering materials (mostly polymers, metals, or ceramics) to create objects [1]. Three-dimensional printing has been applied in many fields, such as the automotive, electronic, and aerospace industries, healthcare, architecture, food, and agriculture [2].

Because 3D printing is patient tailored, it facilitates a precise fabrication and helps to create patient-specific complex geometries, making it attractive for a variety of medical fields. In particular, the advanced implementation of additive manufacturing is observed in maxillofacial surgery [3]. With additive manufacturing, solutions can be found more quickly, and new geometric opportunities can be developed to aid medical applications [4]. Usually, costs can be reduced through 3D printing technology, provided that the right equipment is used and the operator is familiar with the technology. Moreover, using 3D printing technologies reduces manual workload, and, as a result, workers are able to concentrate on other tasks.

However, in dentistry, the implementation of the technology is not yet advanced. The approximately 40-year-old technology is still in its development process and has not entirely diffused yet. An innovation is labeled as diffused when it has been widely adopted, or the conventional method has been replaced with the new technology [5,6]. Awareness, communication, innovativeness, investment and complementary costs are amongst the other key factors in determining the speed of the diffusion process [5,6]. Against this background, the present study aims to identify which determinants need improvement to advance the diffusion process of additive manufacturing.

To allow for a better understanding of the technology, it is important to address the process of additive manufacturing in dentistry. To perform additive manufacturing, a scanning tool and computer software are needed to print the object that will be used as a solution for the patient’s malfunctions. Commonly, an intraoral scanner is used for scanning the oral cavity, which facilitates the substitution of traditional dental impressions. This would enhance the comfort of the patient’s visit. Next, a computer-aided design (CAD) program—commonly Exocad or 3Shape—which enables the realistic construction and drawing of specific shapes is used for designing the desired object. Then, the objects are printed layer by layer and are used to produce a medical product. Currently, this process of printing objects is mostly executed through five different printing technologies—selective laser melting (SLM), selective laser sintering (SLS), digital light processing (DLP), fused deposition modeling (FDM), and stereolithography (SLA) [7]—all of which have different strengths and weaknesses [8].

Although the use of additive manufacturing is increasing in the dental field, it remains unclear why the technology is not used widely in dental practices. Many dentists and dental technicians encounter significant challenges when approaching 3D printing. To identify and rank these obstacles, we conducted a Delphi study with the aim of identifying the factors that pose the greatest challenge to the implementation of 3D printing in dental practices. Our study contributes to the literature by aiding progress in scientific perception relating to 3D printing in prosthodontics and outlining potentials to reduce the obstacles.

## 2. Background

First, a comprehensive literature review was conducted, following a snowball sampling strategy, which led to acquiring the current level of knowledge of the surveyed topic to facilitate the textual preparation for the Delphi method. The literature review, performed by searches of online databases, such as PubMed and Web of Science, demonstrates the manifold uses of 3D printing and its strengths and weaknesses.

In daily practice in dentistry, conventional technology consists of mostly computerized numerical control (CNC) milling of plastics and other materials. CNC milling is progressively being replaced by additive manufacturing, which would be a solution to lower costs, limit production time, and reduce waste [3].

The literature highlights many advantages of additive manufacturing. The main studies on 3D printing in dentistry are to produce custom-made dental implants, crowns, bridges, aligners, bite splints, and print models and dentures [9]. These custom-made productions enable the medical field to reach new levels of care. As a result, their use has increased significantly over recent years [10]. The accuracy of the milling method has been proven to be inferior to the three-dimensional printing method [11]. Furthermore, the benefits of using 3D printing compared to traditional technologies are a higher surface precision and uniform production of crowns [12].

Research on additive manufacturing technologies agree on a variety of advances in 3D printing. Compared to the subtractive conventional technologies, such as CNC milling, the additive manufacturing process, as its name implies, is a process in which the layers are stacked [13]. Therefore, from the usual high amount of waste in subtractive methods, the waste of material in 3D printing technologies can be reduced. Consequently, this creates a progressive impact on advancement in the sustainability of manufacturing [14].

Furthermore, sustainability factors can be enhanced due to local manufacturing. Outsourced production could be restructured and supply chains optimized. A conversion to small inventories is also possible with the technology, since the orders can be fabricated on demand. As a result, transportation, along with its resulting pollution, and costs can be reduced [8,10,14].

As mentioned in the introduction, 3D printing technologies require the acquisition of a scanner and software. The accuracy of the tailor-made products achieved through an intraoral scanner, CAD software, and 3D printer are especially beneficial and cost-effective in small production quantities [15,16].

As mentioned earlier, there are five well-established additive manufacturing technologies that are used in daily practice. SLM and SLS are printing technologies in which a powder material is beamed with a laser such that it softens and hardens again, resulting in the fused desired objects. SLM includes the printing of metal, whereas SLS mostly involves polymers. Additionally, in both technologies, the use of powder fusion printing means that no support structures are needed. Moreover, in the additive manufacturing processes of SLM and SLS, the powder material that has not been used during the printing process can be reused, with a recyclability of up to 95–98% [17]. Another common 3D printing technology is DLP, which is a photopolymer printing technology that creates an object through the transition of a resin into a hardened material through a projector. The main benefit of DLP is its high printing speed and accuracy [8]. FDM is a material extrusion process where commonly a thermoplastic filament is softened through a heated nozzle, which presses the material, layer by layer, onto a build platform. FDM is a highly cost-efficient process. Therefore, many FDM printers are sold for home projects, due to its low costs [18]. With FDM printing, it is usually unnecessary to have a wash and cure process, which makes it easier to use for the broader public. However, FDM currently has a lower resolution compared to SLA printing [13]. Finally, SLA printing is the process of beaming a laser onto photopolymers, which hardens them. This technology has both a high resolution and high accuracy [3].

Despite its advantages, 3D printing has not become a standard technology yet in dentistry. In contrast, in other fields, such as aerospace, research, certification, and production is much further developed. For example, through 3D printing, it is possible to create light models that can replace the heavy-weighted structures and lead to a decreased use of costly and non-environmentally friendly material [6,8,16]. Consequently, the advances of 3D printing in aerospace manufacturing are widely acknowledged [19]. Another increasingly popular application area is architecture [2]. Ngo et al. praise the freedom of design [20] and other scholars add that the range of printable geometric complex structures are immeasurable [21]. Additionally, the history of 3D printing for museum displays and for archaeological studies is noteworthy: By 1992, it was possible to use SLA to create a physical model of a mummy skull. With the aid of the CT scanning technology, it was possible to retrieve data without demolishing the original mummy [22].

However, the literature addresses various challenges of 3D printing technologies; obstacles of 3D printing in the literature mostly refer to the limitations of its materials. Studies have reported that only 10% of 200 products and materials are actually suitable for additive manufacturing [14]. Hence, they are either not fulfilling the capabilities of conventional technologies, need optimization of material, or are understudied in general [23]. Researchers see metal printing as another challenge: First, it is seen as a challenge due to the limitation of material varieties [24]. Second, another concern is the poor heat dissipation, which would be problematic, for example, for dental crowns and bridges [25]. Third, there are the barriers of high initial costs and the long duration of the certification process [16]. Financially, researchers are seeing a decline in expenditures for 3D printing technologies. The high costs of additive manufacturing are usually regarded as more problematic in large supply chains than in small productions. However, limitations are detected when it comes to the actual calculation of the manufacturing costs [26].

To summarize, 3D printing methods with the mentioned materials are promising [23]. However, benefits and challenges encountered with this new technology are still understudied and thus are not tangible yet, and the amount of adequate and certified material is still restricted [17,23]. Moreover, post-processing is complex and must be optimized in the future [3]. Therefore, it is of great importance to conduct further research.

## 3. Methodology

### 3.1. The Ranking-Type Delphi Study

This study addresses the challenges that face the implementation of 3D printing technologies in dental practices in form of a Delphi study design. The research format is a group survey that is especially suitable because several experts were invited to provide their opinions and explain their perspectives in the context of the survey topic [27]. Moreover, Delphi studies have proved to be effective in the healthcare industry, which is based on custom-made applications [28]. To gain an expert opinion consensus, this method has been used in dentistry for many studies, for example, to determine obstacles during implant placement [29] or to form fitting and crucial measures in oral healthcare [30]. The Delphi study method has been used extensively since the 1950s, especially as a forecasting tool [31]. Different from traditional surveys where a randomized selection of individuals takes place, in the Delphi study method, a panel of experts who are qualified to respond to the survey questions are assembled. Further benefits with this research format are that traditional in-person interviews often prevent the experts from speaking truthfully because they want to appear in a certain light in front of the other panelists. Thus, owing to the anonymity between the panelists, the Delphi method ensures that experts are able to express their personal views freely [32].

The ranking-type Delphi study, which has developed from and is therefore a variation of the Delphi research format, was chosen to detect the obstacles that hinder the implementation of 3D printing in dental practices and, especially, pinpoints the affecting factors [33,34]. This variation of the Delphi study is beneficial in studies where limitations of research of the specific topic exist [31].

The survey begins with determining a specific problem associated with the guiding research question. Next, the researcher selects a panel of experts who would be able to answer the guiding question. Then, the researcher develops open-ended surveys that help in examining the defined problem. The goal is to achieve a consensus-based solution to this problem. The responses in the questionnaires are both qualitative and quantitative, which provides the researchers an adequate amount of information, and a reassessment is included in the last round of the survey [33].

In this ranking-type Delphi study, as in all Delphi studies, it is of great importance to select qualified experts in a multi-step process against pre-specified selection criteria [31,33,35]. Okoli and Pawlowski’s [33] “Research guidelines for the Delphi survey technique” facilitated the direction of the research format. Thus, highly experienced dentists, dental technicians, and 3D printing company representatives were recruited to participate based on their professionalism, work experience, and knowledge of dentistry, as well as, for the 3D printing representatives, digitalization expertise and 3D printing capabilities.

### 3.2. Panel

Twelve dentists, six dental technicians, and four representatives of 3D printing companies were contacted via email and telephone, consisting of an invitation to participate and an exposition of research design and topic. In total, 25 experts were contacted, of whom 22 participated, leading to a response rate of 88%. Because none of the participants withdrew from the study, the drop-out rate was 0%.

The participant characteristics are depicted in Table 1. The study sought an equal distribution (50%) of female and male participants to achieve well-balanced results. The average age of the participating experts was 45 years. The average work experience of the experts was 10 years. More than half (54.54%) of the participants were dentists because the study aimed to identify the barriers to the implementation of 3D printing, specifically in dental practices. Because the expertise of dentists did not quite match the expected level of familiarity and expertise regarding 3D printing, six dental technicians and four employees of a 3D printing company were invited to participate to counterbalance the dentists’ unfamiliarity with the digital technology in dentistry.

### 3.3. Data Collection

The survey of these experts was performed in three rounds to detect the challenges of implementation of 3D printing in dental practices. The first round of the study was performed between 16 and 30 June 2021. The second round of the survey was completed by 2 September 2021. The third and final part of the study was performed between 16 and 30 September 2021.

In the first round, the dentists, dental technicians, and 3D printing representatives were asked to list potential challenges in the application of 3D printing in dental practices. The suggestion was to simply brainstorm and name obstacles that hinder the implementation of additive manufacturing in dental practices. For this round, each expert suggested three to five barriers of the implementation on the spot. Then, the 84 identified challenges were categorized, and duplicates were eliminated. Due to the similarities among several answers from the experts, ten obstacles were ultimately forwarded to the second round for ranking. During the second round, the experts ranked the obstacles according to their relevance for more structured feedback. Here, a scale from 1 (=minor-rated obstacle) to 10 (=highest significancy-rated obstacle) was provided to the experts. In the last round, this ranked list, including statistical data, was provided to the experts, and they were asked to modify the ranking assigned by them in the second round, if desired.

## 4. Results

This study aimed to identify the factors that pose the greatest challenge to the implementation of 3D printing in dental practices. During the first round of the study, 10 major challenges were identified as the most critical obstacles to the implementation of additive manufacturing in dental practices (Table 2). These obstacles were related to the manufacturing process, financial aspects, and their comparison to conventional methods. The second and third rounds revealed that the top-ranked obstacles were high training effort for the staff and the lack of familiarity with additive manufacturing among dental experts in general. Manufacturing challenges such as the difficulty of printing with metal, high printing duration, and the occurrence of general material printing difficulties seemed to be less relevant, therefore posing the smallest challenges (first to third position in Table 2). External factors, such as spatial obstacles and investment expenditures, were ranked with a mean score of 4 and 5.5, thus placing fourth and seventh in the overall ranking of obstacles.

The mean score (± standard deviation) of each obstacle changed slightly from the second round to the third round. The average standard deviation score in the first round was 2.68 and declined to an average of 2.64 in the second round. The mean score shifted slightly in the first, ninth and tenth places. For instance, the obstacles “printing machineries limited for metals” declined from 3 to 2.5. The obstacles “high training effort for staff” and “no knowledge of additive manufacturing” showed an increase in its mean score by 0.5, from the second to the third round. However, these changes did not interfere with nor alter the overall placement of the barriers to the implementation of additive manufacturing in dental practices. Another round of the survey, therefore, would have been obsolete because it would not have revealed any significant changes.

## 5. Discussion

The Delphi study demonstrated that the essence of the implementation challenges for 3D printing in dentistry lies in the users’ unfamiliarity with the technology, which seems paradoxical. However, the literature review highlights that, although the technology has existed for more than 40 years, a complete diffusion has not taken place yet [5,6].

While previous research identified the main challenges as being related to manufacturing in certain 3D printing technologies such as SLM [16,24,25], the present study highlights that there exists a more superficial yet crucial factor: the dentists’ lack of knowledge regarding the use of 3D printing in their dental practices. This knowledge is a vital factor necessary to decide which technology should ultimately be used to repair the malfunctioning parts. It is alarming that several dentists—as they stated during the survey—simply send the screenings to their laboratory and wait for the product delivery, without precisely knowing which technologies their laboratory or dental technician uses to create the final products.

Furthermore, the significant difference in the knowledge of additive manufacturing between dental technicians and dentists was worrisome. Although the average standard deviation decreased slightly from the second to the third round, which implies an increase in certainty, the dental technicians involved in this study were more familiar with the technology after all; thus, the standard deviation in all obstacles was relatively high.

Additionally, indications of the lack of knowledge can be observed in the statistical data of the other ranked challenges to implementation. Whereas previous studies [14,23] indicate that a high rate of problems occur within material challenges, for example in the manufacturing of long-term-applications, an intermediate ranking was assigned to this specific problem in the present study, which suggests that either profound knowledge of the disadvantages of the technology’s material was not present within the expert pool or the innovation has improved during the diffusion process. According to Rosenberg, the enhancement of the technology while adapting to its new environment is possible, as innovation diffuses [5,36,37].

High initial costs constitute another important obstacle. A 3D printer, CAD software, intraoral scanner, and material to print dental models or to create bite splints would cost a dental practice approximately EUR 22,900 [38]. In the present study, the initial costs were ranked as the seventh highest obstacle. Investment costs are therefore not an obstacle that the dentists seem to easily overlook, consistent with previous findings [16,26]. However, in the survey, the participants stated that, if dentists and co-workers had profound knowledge of the technology, they would not disregard an investment in a “printing kit”. An investment in a technology that one is not familiar with is rarely observed, especially in the medical industry. Therefore, if substantial knowledge of the technology is established and advanced in the dental practices, and advantages would outweigh financial distress, diffusion would progress more rapidly [5].

Additionally, the cost barrier is closely associated with the obstacle “not practical for small practices,” which ranked sixth in this study. This factor also exhibited the highest standard deviation in the second and third rounds, which can be explained by the fact that the expert-pool consisted of both experts who own a small practice and those who own a larger practice and were, therefore, optimistic, about investing in additive manufacturing in a small practice. Practices were considered as small when they included one to three dentists and their team. Thus, larger practices included four or more dentists. Experts with small practices were further worried that complementary costs, in the form of training the staff and adding facilities for manufacture, would outweigh the profitability.

In summary, the high rank of the obstacle “no knowledge of additive manufacturing” highlights the need to advance the diffusion process of the technology—not only in the awareness stage but also regarding knowledge transfer and cost reduction.

### 5.1. Implications

To achieve a more profound understanding of 3D printing in dental practices, the awareness of the innovation must first be expanded. The Bass model, a theoretical framework of marketing, suggests that it is essential to distribute awareness through mass media early on and to follow-up with an increase in interpersonal communication to achieve a more rapid growth in implementation [5,39].

Furthermore, there is an unquestioned need for training employees. An offer for several training opportunities for the staff is important to ensure the advancement of additive manufacturing in dental practices. An improved strategy for sales and distribution could help in the advancement of education. The distribution of 3D printers is usually achieved by 3D printing companies or resellers, such as dental depots. When sales are completed by a 3D printing company, the customer, i.e., the dentist or dental technician, receives a service package. Such a package can entail a 90 min training on how to operate the printer and to become familiar with the material. Not only is a 90 min transfer of knowledge not sufficient to acquire a profound understanding but it is also a service not necessarily provided in all 3D printer sales. Consequently, this study suggests the requirement of a beneficial service. Additionally, an expansion of the duration of supervision and support would be necessary to result in efficient operating of the 3D technology, higher customer satisfaction, and customer retention, as well as a potential growth of the customer base. Employees who are able to apply skills rapidly to new innovations and processes and are trained in fundamental technical information need long-term training and retraining [40].

Furthermore, sales, solely via medical consulting services, would be recommended. Sales representatives of additive manufacturing need to be well trained. Additionally, resellers need to be well grounded in the technology and qualified to sell 3D printers in the dental industry.

An important discovery of the study arose in terms of obstacles within the internal knowledge transfer between dental technicians and dentists. It would be helpful if the different departments conducted technical and strategical meetings to improve the overall understanding of digital advancement, technological and material updates, and management strategies. This would ensure a balanced knowledge transfer throughout the units and prevent crucial errors in planning, management, and production. Relocating the manufacturing from external to internal production processes would further facilitate a quicker in-company knowledge transfer.

Further implications entail the assessment of 3D-printed technology and its printed products as medical products. With medical products comes the responsibility to follow strict medical device regulations (MDR). As its name implies, MDR regulates the requirements of the sale of medical devices. The health organization, i.e., dental practices, need to follow, amongst other rules, Article 5(5b) of the MDR regulations: “manufacture and use of the devices […] under appropriate quality management systems” [41], which means that the use of medical products requires detailed documentation of printing processes and risk assessment. This would further enhance the training of technology processes of the staff and improve the work flow with the material. As a result, the existing knowledge gaps in additive manufacturing can be closed more quickly.

While this study uncovered the knowledge gaps of AM in dental practices, the findings of this study provide insights to addressing these gaps and advancing the familiarity with 3D printing in the future. Closing the knowledge gaps through training programs is an important aspect, yet a continuation and personalization within the training is equally essential.

### 5.2. Limitations and Future Research

The limitations of the study were mainly related to the COVID-19 pandemic and its accompanying restrictions. Thus, it was challenging to reach participants, schedule telephone appointments and conduct the second and third rounds of the survey. However, through persistency, as many participants as possible were reached. While the common number of experts, according to Delphi study norms, was attained, a higher number of experts in another survey format would be interesting to explore in future research to analyze the assessments of a larger community [27]. The choice of the expert pool led to a high standard deviation, which could be decreased by assembling an exclusive panel. Due to the higher expert knowledge of dental technicians in 3D printing, future research could therefore focus solely on dental technicians.

Another future research recommendation entails analyzing the challenges in implementing 3D printing regarding resin processing and cost comparison to conventional milling technologies. The process of closing the existing knowledge gaps should be further analyzed through carefully scrutinizing the training of dentists and dental technicians within particular steps during manufacture, regarding software or operating scanners. The education processes could potentially be analyzed in future follow-up studies, which would serve to further uncover barriers to implementation.

## 6. Conclusions

The three-round ranking-type Delphi study reveals the barriers to the implementation of 3D printing in prosthodontics. Twenty-two participants with relevant professional expertise highlighted the difficulties that hinder the realization of additive manufacturing in dentistry, particularly in dental practices. The 10 most important obstacles were identified and ranked on a 1–10 scale, according to their relevance. The findings suggest that the familiarity with 3D printing within dental practices must be improved immensely to successfully integrate additive manufacturing technology into dental practices. The study’s findings suggest that, to enhance the rapid diffusion of this innovation, awareness of the technology, training of distributers, resellers, and employees have to be improved and should become priorities. Consequently, many aspects need further improvement if the use of this innovative technology is to become widespread in the future.

## Figures and Tables

**Table 1:** Panel Description.

Gender	Female	11 (50.00%)
	Male	11 (50.00%)
Age (years)	<31	2 (9.09%)
	32–41	7 (31.28%)
	42–51	7 (31.28%)
	52–61	6 (27.27%)
	>62	0 (0.00%)
Work experience	<6	4 (18.18%)
	6–10	8 (36.36%)
	>10	10 (45.45%)
Occupation	Dentist	12 (54.54%)
	Dental Technician	6 (27.27%)
	3D Printing Representative	4 (18.18%)

**Table 2:** Ranked obstacles of second and third survey round.

Second Round				Third Round			
Place	Obstacle	$\bar{r}$	σ	Place	Obstacle	$\bar{r}$	σ
1	printing machineries limited for metals	3.00	2.98	1	*printing* *machineries limited for metals*	*2.50*	*2.9*
2	applications not practical to print	3.50	2.36	2	applications not practical to print	3.50	*2.34*
3	print duration too long	3.50	2.58	3	print duration too long	3.50	*2.54*
4	no facilities for manufacturing	4.00	2.36	4	no facilities for manufacturing	4.00	*2.35*
5	not applicable for long-term-applications	4.00	2.61	5	not applicable for long-term-applications	4.00	*2.59*
6	not practical for small practices	4.50	3.23	6	not practical for small practices	4.50	*3.12*
7	high costs	5.50	2.91	7	high costs	5.50	*2.75*
8	prefers conventional methods, no interest	6.00	2.42	8	prefers conventional methods, no interest	6.00	*2.39*
9	high training effort for staff	6.50	2.58	9	*high training effort for staff*	*7.00*	*2.69*
10	no knowledge of additive manufacturing	8.00	2.76	10	*no knowledge of additive manufacturing*	*8.50*	*2.72*

$\bar{r}$: average rank; σ: standard deviation. Shifts are depicted in italics.

## Data Availability

The data presented in this study are available on request from the first author.
